# Cheetahs have a stronger constitutive innate immunity than leopards

**DOI:** 10.1038/srep44837

**Published:** 2017-03-23

**Authors:** Sonja K. Heinrich, Heribert Hofer, Alexandre Courtiol, Jörg Melzheimer, Martin Dehnhard, Gábor Á. Czirják, Bettina Wachter

**Affiliations:** 1 Department of Evolutionary Ecology, Leibniz Institute for Zoo and Wildlife Research, Alfred-Kowalke-Str. 17, 10315 Berlin, Germany; 2 Department of Evolutionary Genetics, Leibniz Institute for Zoo and Wildlife Research, Alfred-Kowalke-Str. 17, 10315 Berlin, Germany; 3 Department of Reproduction Biology, Leibniz Institute for Zoo and Wildlife Research, Alfred-Kowalke-Str. 17, 10315 Berlin, Germany; 4 Department of Wildlife Diseases, Leibniz Institute for Zoo and Wildlife Research, Alfred-Kowalke-Str. 17, 10315 Berlin, Germany

## Abstract

As a textbook case for the importance of genetics in conservation, absence of genetic variability at the major histocompatibility complex (MHC) is thought to endanger species viability, since it is considered crucial for pathogen resistance. An alternative view of the immune system inspired by life history theory posits that a strong response should evolve in other components of the immune system if there is little variation in the MHC. In contrast to the leopard (*Panthera pardus*), the cheetah (*Acinonyx jubatus*) has a relatively low genetic variability at the MHC, yet free-ranging cheetahs are healthy. By comparing the functional competence of the humoral immune system of both species in sympatric populations in Namibia, we demonstrate that cheetahs have a higher constitutive innate but lower induced innate and adaptive immunity than leopards. We conclude (1) immunocompetence of cheetahs is higher than previously thought; (2) studying both innate and adaptive components of immune systems will enrich conservation science.

Conservation science is a discipline that can help to slow down global biodiversity loss^1^,^2^. The integration of life history theory and other facets of evolutionary ecology into conservation science has the potential to provide new conservation management tools^3^. This is because an evolutionary approach uses a theoretical or empirical framework which provides testable predictions on the diversity of physiological responses to disturbances in individuals under natural selection^4^. With such knowledge, conservation management activities can be derived with more predictable outcomes than with the alternative trial and error approach^5^.

One of the strongest natural selection pressures are pathogens that challenge the immune system of individuals and can lead to diseases and in many cases to death^6^,^7^. The usually highly polymorphic multigene family of the major histocompatibility complex (MHC) is part of the adaptive immunity and encodes key receptor molecules that recognise and bind foreign peptides for presentation to immune cells^8^. It is generally assumed that resistance to infection is more effective the more MHC loci and alleles exist in a host individual^9^. This is because a heterozygous individual with many loci is more likely to detect and respond adequately to a wider range of pathogens which will increase its fitness than an individual which is homozygous at one or more loci^10^. The more alleles are present in a population, the more it is likely that an individual will be heterozygous. Thus, balancing selection is likely to maintain high allelic diversity at the MHC because of selection pressures exerted by infections^11^,^12^,^13^.

There is considerable evidence that high variability of MHC genes improves pathogen resistance. This comes mostly from humans or animals under laboratory conditions^14^, although recent studies considered free-ranging populations (for example refs 15, 16, 17, 18, 19, 20). The evidence is less clear on whether low MHC variability necessarily reduces population viability^21^. Examples of populations with low MHC variability and low susceptibility to diseases were described for, e.g., Chillingham cattle (*Bos taurus*)^22^, North American and European moose (*Alces alces*)^23^,^24^ and mountain goats (*Oreamnos americanus*)^25^. There are also examples of populations with high MHC variability and a high susceptibility to diseases, as in bighorn sheep (*Ovis canadensis*)^26^. These studies provide evidence in contrast to the expectation that low MHC variation results in an impaired immune response and vice versa^12^,^27^,^28^.

With an evolutionary ecology approach, one possible explanation of these constellations would be that there would have been selection pressure to strengthen other components of the immune system to provide an adequate compensatory immune response^29^,^30^. Thus, if a reduction in immunogenetic variability in a species impairs an immune component such as adaptive immunity, other components of the immune response might compensate any reduction in functionality^30^. This suggests that components of the immune system have the potential to interact, inhibit or compensate each other in species-specific ways, as has been theoretically discussed and empirically demonstrated^29^,^30^,^31^, and that similar levels of protection against pathogens may be accomplished by different combinations of protective systems. Compensatory responses could, for instance, be channeled through a rise in the energetic investment in non-impaired immune components, or be the consequence of the evolution of increased genetic variation in non-impaired immune components.

To assess the potential diversity in investment and the overall immunocompetence of individuals, it is important to simultaneously measure several components of the immune response^32^. Here we combine functional assays and measurements of several effectors of the immune system in a species with relatively low genetic variability, the cheetah (*Acinonyx jubatus jubatus*), and compare them with measures from a species with relatively high genetic variability, the African leopard (*Panthera pardus pardus*) from sympatric free-ranging populations in Namibia.

Cheetahs exhibit a relatively low genetic variability^33^,^34^, including the loci of the MHC^35^,^36^, although this is actually higher than previously thought^37^. It was previously assumed that the consequence of this relatively low genetic variability was a high susceptibility to diseases and a poor reproductive performance^33^,^34^,^35^,^38^. However, such susceptibility and poor performance have only been reported from cheetahs in captivity^39^,^40^. Free-ranging cheetahs showed no clinical or pathological evidence for diseases, even when tested seropositive for several infectious diseases^41^, and they successfully reproduced^42^. The findings in captivity were later shown to be a consequence of unfavorable husbandry conditions and breeding management rather than the relatively low genetic variability^43^,^44^.

The African leopard is sympatric with the cheetah in the same habitat in Namibia. Individuals of both species live solitarily or in small groups consisting of mothers with their offspring. In cheetahs, independent litter mates or unrelated adult males may also form long-term coalitions of two to three animals^45^,^46^. Cheetahs and leopards generally hunt the same prey animals^46^,^47^, although the leopard has a wider dietary breadth and also scavenges regularly. Leopards might therefore be exposed to the same pathogens but at higher contact rates than cheetahs. In contrast to cheetahs, leopards exhibit a relatively high genetic diversity; the leopard in Namibia is the subspecies which displays the highest diversity in mitochondrial DNA and microsatellite markers of all big cat species^48^,^49^. Consequently, the MHC diversity of Namibian leopards is higher than that of cheetahs^50^. Thus, the MHC dependent adaptive immune response of cheetahs might be weaker than that of leopards^51^. If immune components can compensate each other, e.g. through an increase in energetic investment^29^,^30^, we expect Namibian cheetahs to invest more in the innate immune response than Namibian leopards.

Here we report the results of our measurements of humoral immunity generated by the three major components of the immune system, by assessing (1) adaptive immunity, (2) induced innate immunity and (3) constitutive innate immunity. Adaptive immunity is highly specific towards pathogen recognition and can only fight against a pathogen that was encountered previously and for which a specific antibody or T-cell was developed. The predominant antibody isotype in mammals is immunoglobulin G (IgG)^52^, which was chosen as a representative for adaptive immunity in this study. Induced innate immunity, most importantly the acute phase response, is unspecific towards pathogen recognition and thus can react quickly in case of a challenge. It serves to restore homeostasis in the host and therefore increases in concentration shortly after tissue injuries or inflammations, or the experience of short-term stressors^53^,^54^. Serum amyloid A (SAA) is the most important acute phase protein in domestic cats^55^,^56^,^57^,^58^ and in cheetahs^59^. Thus, SAA was chosen as a representative for induced innate immunity. Constitutive innate immune effectors provide a rapid first line of defense against intruders. The bacterial killing capacity of plasma and serum is primarily mediated by the complement and other antibacterial proteins and is a functional measurement of the constitutive innate immunity. Lysozyme is a major part of the constitutive innate antibacterial immunity which acts by digesting peptidoglycans of bacterial cell walls, especially of gram-positive bacteria^60^. The haemagglutination/haemolysis assay is a method that quantifies two interrelated parts of the constitutive innate immune system, natural antibodies (haemagglutination titer) and the complement system (haemolysis titer). Natural antibodies recognize epitopes of various antigens and initiate the complement system, a group of proteins that trigger a signaling cascade which finally leads to pathogen lysis^61^,^62^. We therefore chose the bacterial killing assay, the lysozyme assay and the haemagglutination/haemolysis assay to characterize constitutive innate immunity.

Because morphological, immunological and endocrinological parameters may be affected by differences in allostatic load (‘stress’)^63^,^64^, we measured the impact of trapping cheetahs and leopards in box traps in terms of their glucocorticoid concentrations in a quasi-experimental setup. This permitted us to take into account possible differences in allostatic load between individuals or species^65^,^66^.

## Results

**Figure 1:**
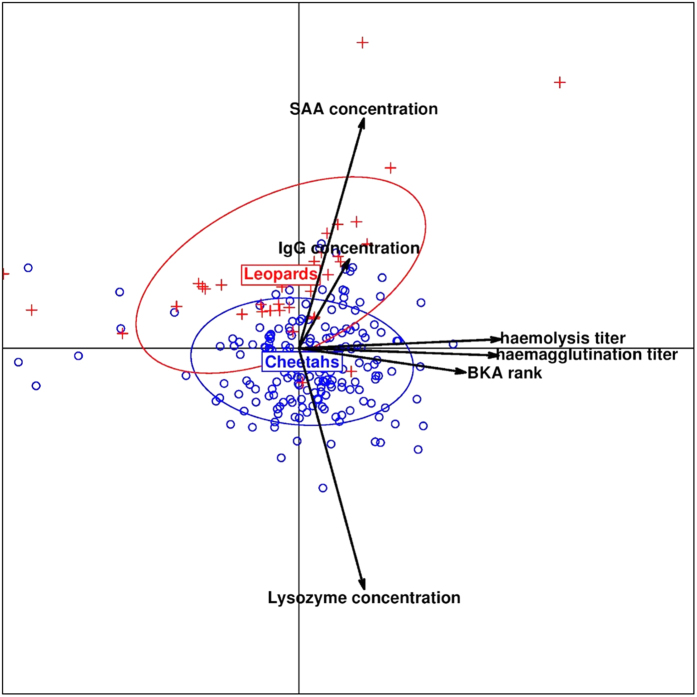
Immune differences between cheetahs and leopards. Position of all cheetahs (small circles) and leopards (plus signs) projected into the space defined by the first two principal components (PC1 in x-axis and PC2 in y-axis) of a principal component analysis performed on all six immune parameters. Together PC1 and PC2 capture nearly 64% of the total variance. Arrows represent the contribution of each immune parameter to PC1 and PC2. For each species, 1.5 inertia ellipses are depicted.

We first reduced the dimensionality of the data for an overall comparison of the immunity of cheetahs and leopards by summarizing the six immune measurements into the two first principal components (PC) of a principal component analysis (PCA, Fig. 1), as suggested by Buehler *et al*.^67^. To maximize the use of information contained within the data, we used an expectation-maximization algorithm to perform multiple imputation for incomplete data (see methods) before running the PCA. We ran PCAs on both the larger data set with imputed data (Model 1) and the original raw data set (Model 3) and found that the outcome was qualitatively very similar (see Supplementary Results). We therefore report here the results for the complete data set with imputed data.

PC1 captured 40.1% of the total variance in immune measurements and mainly reflected constitutive innate immunity, because three of the four variables for constitutive innate immunity aligned along the x-axis, which represents PC1, in Fig. 1 – these were bacterial killing assay (BKA) ranks, haemagglutination titer and haemolysis titer (see Table S3 for PCA loadings). Table S2 summarizes the logistic regression model that assesses the reliability of predicting species identity from the PCA. Cheetahs presented significantly higher scores (mean PC1 value: 0.042) than leopards (mean PC1 value: −0.230) on the first principal component (logistic regression, likelihood ratio test (LRT) = 7.45, *df* = 1, *P* = 0.006).

PC2 captured 23.8% of the total variance in immune measurements and mainly reflected the induced innate and adaptive immunity in terms of SAA and IgG concentrations, but also the constitutive innate immunity in terms of lysozyme concentrations (Fig. 1; Table S3). On PC2, cheetahs (mean PC2 value: −0.237) presented significantly lower scores than leopards (mean PC2 value: 1.299, logistic regression, LRT = 63.86, *df* = 1, P < 0.001).

There were no differences in immune values between the sexes in cheetahs (LRT = 0.19, *df* = 2, P = 0.91) or in leopards (LRT = 1.81, *df* = 2, P = 0.40), nor between adult and sub-adult cheetahs (LRT = 0.39, *df* = 2, P = 0.82) or leopards (LRT = 4.03, *df* = 2, P = 0.13).

**Figure 2:**
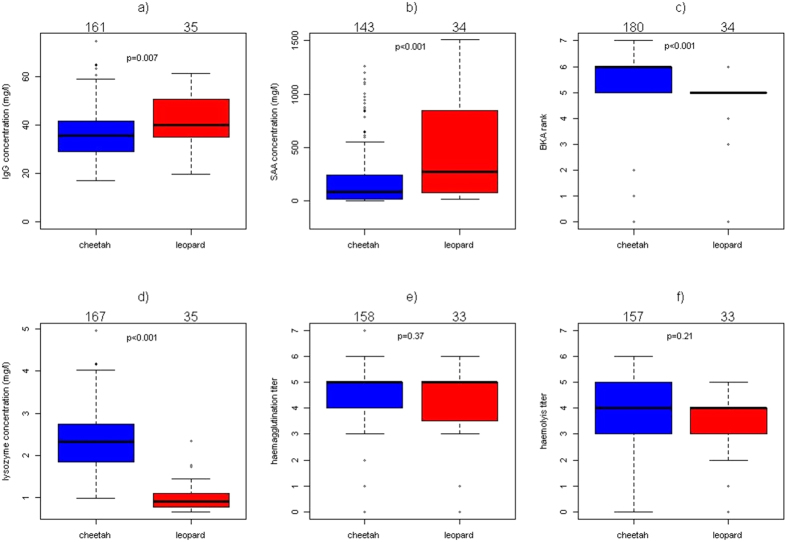
Pairwise comparison of immune parameters of cheetahs with leopards. (**a**) Immunoglobulin G concentration (**b**) Serum amyloid A (SAA) concentration (**c**) Bacterial killing assay (BKA) ranks (**d**) Lysozyme concentration, (**e**) Haemagglutination titer (**f**) Haemolysis titer. (**a**) is part of the adaptive immunity, (**b**) of the induced innate immunity and (**c**–**f**) are part of the constitutive innate immunity. Boxplots depict medians with 25% and 75% quartiles, *P*-values are indicated above the bars and samples sizes above the graphs.

We then characterized differences between cheetahs and leopards by performing pairwise comparisons for all immune variables (Fig. 2a–f) using only the actual measurements, i.e., without imputing missing values. In terms of adaptive immunity and induced innate immunity, cheetahs (mean_cheetahs_ = 37.2 ± 11.0 mg/l) had lower IgG concentrations than leopards (mean_leopards_ = 42.1 ± 10.5 mg/l, Mann-Whitney U-test, *W* = 1998.5, *N*_*cheetahs*_ = 161, *N*_*leopards*_ = 35, *P* = 0.007). Cheetahs (mean_cheetahs_ = 213.9 ± 303.1 mg/l) also had lower SAA concentrations than leopards (mean_leopards_ = 605.3 ± 842.6 mg/l, *W* = 1462, *N*_*cheetahs*_ = 143, *N*_*leopards*_ = 34, *P* < 0.001). Concerning constitutive innate immunity, cheetahs had higher BKA ranks than leopards (*W* = 4427.5, *P* < 0.001, *N*_*cheetahs*_ = 180, *N*_*leopards*_ = 34), with median ranks of 6.0 and 5.0, respectively, indicating a bacterial killing capacity of cheetahs which was twice as high as that of leopards. Serum lysozyme concentrations in cheetahs (mean_cheetahs_ = 2.34 ± 0.64 mg/l) were higher than in leopards (mean_leopards_ = 1.01 ± 0.37 mg/l, *W* = 5658, *N*_*cheetahs*_ = 167, *N*_*leopards*_ = 35, *P* < 0.001). There was no difference between species in haemagglutination titer (*W* = 2850.5, *P* = 0.37, median_cheetahs_ = 5, median_leopards_ = 5, *N*_*cheetahs*_ = 158, *N*_*leopards*_ = 33) or haemolysis titer (*W* = 2927.5, *P* = 0.21, median_cheetahs_ = 4, median_leopards_ = 4, *N*_*cheetahs*_ = 157, *N*_*leopards*_ = 33). Note that all of the aforementioned significant pairwise comparisons would remain significant if p-values were corrected for multiple testing using the Bonferroni procedure or more powerful alternatives to this method.

Serum cortisol concentrations in cheetahs (mean_cheetahs_ = 19.8 ± 18.3 ng/ml) were significantly lower than in leopards (mean_leopards_ = 57.0 ± 18.1 ng/ml, *W* = 338, *N*_*cheetahs*_ = 166, *N*_*leopards*_ = 34, *P* < 0.001), suggesting a higher allostatic load caused by the capture and handling procedure of the latter. There was no difference in the levels of injuries incurred by both species in the box traps (levels of injuries ranged from 1 to 4, Pearson’s Chi-squared-test, *Χ*^*2*^ = 2.67, *df* = 3, *P* = 0.446, *N*_*cheetahs*_ = 182, *N*_*leopards*_ = 32). Cortisol concentrations were influenced by age in cheetahs (Mann-Whitney-U-Test, *W* = 1278.5, *P* = 0.038, cortisol_adult_ = 18.8 ± 17.6, cortisol_subadult_ = 25.0 ± 19.2), but not in leopards (Mann-Whitney U-test, *W* = 53.5, *P* = 0.18, cortisol_adult_  = 55.5 ± 17.7, cortisol_subadult_ = 66.4 ± 19.0). Cortisol concentrations were not influenced by sex in leopards, (Mann-Whitney U-test, *W* = 145.5, *P* = 0.97, cortisol_males_ = 56.7 ± 20.3, cortisol_females_ = 57.3 ± 15.9) but were influenced by sex in cheetahs (Mann-Whitney-U-Test, *W* = 3131, *P* = 0.009, cortisol_males_ = 17.9 ± 16.2, cortisol_females_ = 26.1 ± 21.9).

When correcting for the potential influence of cortisol concentrations and then summarizing the modified immune parameters through a PCA (Model 2), we obtained qualitatively very similar results as those described above for the PCA for Model 1 (see Supplementary Results and Supplementary Table S3). We used a logistic regression with PC1 and PC2 as input to check whether both species could be reliably distinguished by immune parameters and showed that this was the case (Supplementary Results). When correcting for the possible effect of cortisol concentrations in the original, non-imputed dataset (*N*_*cheetahs*_ = 80, *N*_*leopards*_ = 29) before summarizing the modified immune parameters through a PCA (Model 4), we obtained qualitatively very similar results (see Supplementary Results and Table S3) as those from the PCA where no such correction took place (see Model 3 above).

## Discussion

In this study we characterize the immunity of free-ranging Namibian cheetahs and demonstrate that despite presenting a relatively low MHC variability as previously described for this population^36^ other parts of their immune response are not as impaired or reduced as previously thought. Our results show that cheetahs have a higher constitutive innate immune response than leopards, although their induced innate and adaptive immune response is lower. Thus, cheetahs might compensate the potential lack of immunocompetence in the adaptive immune system caused by their low MHC variability with a competent humoral constitutive immune system. If so, our findings could be an explanation as to why free-ranging cheetahs do not suffer from infectious diseases, particularly when tested seropositive for several virulent feline viruses, canine distemper virus and rabies virus^41^,^68^,^69^,^70^. The presence of antibodies previously measured in our cheetah study population^41^,^68^,^69^,^70^ demonstrates exposure to pathogens, and subsequent monitoring of the life histories of our individually recognized study animals demonstrate their long-term survival after exposure.

The effect of allostatic load is known to differ between species and type of immune components measured. The full acute phase response is an example of an immune component that can be triggered by a short-term stressor^53^. Bacterial killing capacity was not influenced by stress in common noctules (*Nyctalus noctula*)^65^, red knots (*Calidris canutus*)^71^, bluecrowned motmots (*Momotus momota*) and claycolored robins (*Turdus grayi*)^66^ but was depressed by acute stress in three other bird species^66^. The level of natural antibodies (assessed by the hemagglutination assay) is not sensitive to stress^61^ and accordingly, hemagglutination and hemolysis assays were not affected by handling stress in red knots (*Calidris canutus*)^71^. We used alternative models (see Supplementary Material) in which we corrected for the potential influence of allostatic load on immune measurements in both the full, imputed data set and the original raw data, and obtained results very similar to the original models. We therefore conclude that our results were not influenced by the differences in allostatic load observed between species.

Immunological measurements may also vary with the time of day^72^ or month of year^72^,^73^,^74^. Our traps were open throughout the year and cheetahs and leopards entered the traps at any day of the year. Animals usually entered the traps between dusk and dawn and therefore were mostly sampled in the morning. Deviation from this sampling scheme was random for the species, thus no systematic bias in our data or results were expected in this respect.

MHC variability primarily affects the functionality of the adaptive immune response^51^. In our study, the adaptive part of the immune system was assessed by the predominant circulating antibody isotype in mammals, the concentration of IgG^52^. Lower levels of IgG have been associated with recurrent opportunistic bacterial infections in domestic animals^75^ and lower overwinter survival in barn swallows^76^. Thus, higher levels of IgG can be regarded as a higher protective level of humoral adaptive immunity and a greater allocation of resources to this part of immunity^77^. Cheetahs had significantly lower concentrations of IgG than leopards. Compared to leopards, this might indicate a reduction of investment by cheetahs into adaptive immunity associated with their lower MHC diversity^36^,^50^. However, IgG is also produced in response to dietary and environmental antigens^77^. Higher concentrations of IgG of leopards might therefore also be a consequence of dietary differences between the two species, as leopards have a greater dietary breadth than cheetahs and are frequent scavengers. They might therefore encounter a larger abundance of pathogens and parasites, including repeated contact with pathogens colonizing carrion^78^, both suggested to increase selection pressure on adaptive immunity. In contrast, cheetahs hardly ever scavenge and predominantly feed on freshly killed meat^46^,^78^.

Regarding induced innate immunity, we detected lower concentrations of SAA in cheetahs than in leopards, even though it can increase 10 to 50 fold during illness in cheetahs^55^,^59^. SAA is the main acute phase protein in domestic cats. Concentrations of acute phase protein rapidly increase when inflammation occurs and rapidly decrease after elimination of the inflammation^59^, or in response to a short-term stressor^58^. Thus, leopards either suffered from acute inflammation at the time of capture or, perhaps more likely, capture and immobilisation induced an increase in cortisol concentration followed by an increase in SAA concentration. Leopards also exhibited higher cortisol concentrations, although both cheetahs and leopards were exposed to the same capture and handling procedures. We interpret these findings to suggest that leopards might respond more strongly to such short-term challenges than cheetahs and as a consequence mount a stronger acute phase response when captured and handled than cheetahs.

The constitutive innate immunity is a first line defense against pathogens and can be activated quickly in case of infection. The bacterial killing capacity determines the ability to remove a bacterial pathogen that could be encountered in the wild and thus is (1) a functional test of the immune system, and (2) provides an environmentally relevant immune response^79^. In wild birds, mimicking a bacterial infection^80^,^81^ or manipulating parasite loads^82^ resulted in an increase in bacterial killing capacity, providing good evidence for the value of this assay. The higher bacterial killing capacity and lysozyme concentration of cheetahs indicates a higher investment into this immune branch by cheetahs than by leopards. Studies of natural antibody titers in mammals are scarce, but a study on free-ranging herbivores revealed large differences between species^83^.

A focus on innate immunity was suggested for insular birds with lower genetic variability than continental birds^30^. As in island populations, at least two scenarios explain a shift of investment towards constitutive innate immunity in cheetahs. Either the relatively low genetic variability of cheetahs is a characteristic of the species and cheetahs invested throughout their evolutionary history more into their constitutive immunity than leopards, or cheetahs lost their previous adaptive immunity as a result of a demographic bottleneck(s) and in response changed their immune investment.

The first scenario is consistent with a recent genetic study on phylogeography and divergence time of extinct and extant African and Asian cheetah populations^37^. Previously, the relatively low MHC variability of cheetahs was thought to be the result of a first demographic bottleneck at the end of the late Pleistocene, approximately 10,000 years ago, and a second one as a result of direct and indirect anthropogenic actions in the past 200 years^33^. However, many mammal species went through a demographic bottleneck at the end of the late Pleistocene, including the leopard^84^, which has a high genetic variability also at the MHC genes^48^,^49^,^50^.

It was also suggested that a strong adaptive immunity may impede the evolution of genetic disease resistance in mammals by reducing selection pressure on the evolution of innate resistance traits^85^. Genetic disease resistance provides a structural basis to prevent particular pathogens to enter and harm a host. Therefore, a weaker^30^ adaptive immunity may improve the chance that alleles for genetic disease resistance go to fixation in a population^85^. As adaptive immunity has substantial energetic costs^86^, a strong innate immunity or genetic disease resistance should reduce selection pressure on strengthening adaptive immunity, which may have occurred in the cheetah^43^.

Alternatively, if cheetahs had a higher genetic variability in adaptive immunity in the past, genetic drift may have been responsible for the possible loss of variability^87^. This is likely if balancing selection on MHC alleles is not particularly strong, e.g., if the benefits did not outweigh the high costs of MHC diversity and expression^88^. For instance, adaptive immunity has been suggested to be more important for social than solitary species because the probability of being repeatedly exposed to the same pathogens rises with higher contact rates with conspecifics, a core feature of group life^86^,^89^. As cheetahs are a solitary species^46^, they would therefore be under less selection pressure to do so. If so, the most frequent MHC allele may get fixated and drift may prevent the fixation of subsequent mutations, keeping MHC diversity at a low level^90^.

Overall, our results are compatible with a focus of immune investment by cheetahs on constitutive innate immunity rather than adaptive immunity when compared to leopards. However, this study compares the immune profiles of only two species. Different life histories in different species may drive immune profiles into different directions and thus the detected differences may have been caused by other factors we are unaware of or which are not linked to each other.

Although the maintenance of the immune system is an important aspect of disease resistance and thus contributes to the survival of the individual, each of its parts has its own inherent costs and protective values^91^. An evolutionary view of the immune system derived from life history theory would therefore argue that individuals have to trade-off these costs with other life-history traits such as growth and reproduction^88^,^92^,^93^,^94^. Cheetahs would therefore be expected to have their species-specific combination of immune defenses and other protective systems optimized for their ecological niche and life history. Our results suggest that such a protective immune phenotype is achieved in cheetahs by investing in constitute innate immunity, whereas leopards focus on the induced branches of the immune system.

The constitutive innate immunity is regarded as relatively cheap, whereas the induced innate immunity has high energetic and potentially pathological costs^88^. Therefore, cheetahs may invest more than leopards in the cheaper immune parts, perhaps because they have fewer energy reserves in form of fat depots^95^. Cheetahs have a slim body built for high speed chases to catch prey^96^ where maximum maneuverability^97^ is essential, but they lack the power and body mass to defend kills from other carnivore predators. The costs of induced adaptive immune responses which generate antigen-specific antibodies are assumed to be comparably low, although they involve high developmental costs generated by complicated and time-consuming lymphocyte diversification processes^88^. These processes are mostly restricted to the developmental period of the animal and require a substantial investment of energy and nutrients during ontogeny^98^. Cheetahs might not have the energy available to invest so heavily in this immune branch during development, since cheetah females usually raise litters of three to six cubs, whereas leopards usually only raise one or two^46^.

Regardless of whether the low MHC variability of cheetahs is a consequence of the loss of high variability through a population bottleneck, genetic drift or selection against high variability, this study demonstrates high investment (lysozyme concentration) and functionality (bacterial killing capacity) of the cheetah’s constitutive innate immune system. It has been suggested that half of the genetic variability for resistance to infections is attributable to non-MHC genes^99^. We suggest that the investment of free-ranging cheetahs and leopards in different immune branches might be equally successful in this habitat and that the immunocompetence of cheetahs might be higher than previously thought.

Our study highlights the importance of an evolutionary approach to the immune system derived from life history concepts, which argues that different parts of the immune system may evolve to cope with species-specific challenges within the trade-offs imposed by the resources available to an organism. This study might inspire new research that test predictions derived from our hypothesis for additional species. We used two large sympatric mammals that contrast in one important immune component, MHC variability. Other species also need to trade-off their resource allocations, thus a phylogenetic approach to compare immune investment across additional species would be useful. This requires that future studies simultaneously measure many or all parts of the immune system and do not limit themselves to the induced adaptive immune response (i.e., MHC variability). This would help to elucidate species-specific and/or habitat and environment-specific adaptations of the immune system.

## Material and Methods

### Study animals

Between 2002 and 2013 we captured 197 (49 female, 148 male) adult (>2 years of age) and sub-adult (>1–2 years of age) free-ranging cheetahs and 36 (19 female, 17 male) adult (>2 years of age) and sub-adult (>1–2 years of age) free-ranging leopards in box traps on farmland in east-central Namibia and immobilized them as previously described^68^. The animals were captured throughout the year. Once captured, they were kept in the box traps in the shade for several hours or overnight until the research team met at the box trap which was normally in the morning hours. The animals were immobilized with a dart gun and blood samples were taken between 20 min and 35 min after darting. After approximately 45 min to 60 min, the animals were given an antidote and observed until they had fully recovered from anaesthesia. From animals that were captured and sampled more than once (*N* = 30 cheetahs), one sample was randomly selected in order to avoid pseudo-replication in the analyses. The proportions of males (75.1% in cheetahs, 47.2% in leopards) differed between the species (Fisher’s exact test, *P* = 0.001), whereas the proportion of sub-adults (15.2% in cheetahs, 16.6% in leopards) did not (Fisher’s exact test, *P* = 0.61).

Animals in traps often acquired small injuries caused by their behavior in the trap. We recorded injuries and assessed the level of injuries as level 1 if they presented no injuries or only had old scars, as level 2 if they presented bloody claws, a bloody nose or one small abrasion, as level 3 if they presented several abrasions, small wounds or one abrasion and a bloody nose or bloody claws and as level 4 if they presented larger abrasions or larger wounds or injuries.

All experimental procedures described in the material and methods were approved by the Internal Ethics Committee of the Leibniz Institute for Zoo and Wildlife Research (IZW, Berlin, Germany) (permit number: 2002-04-01) and the Ministry of Environment and Tourism of Namibia (permit numbers: 525/2002, 700/2003, 764/2004, 939/2005, 1089/2006, 1194/2007, 1300/2008, 1392/2009, 1514/2010, 1514/2011, 1689/2012, 1813/2013), and all experiments were carried out in accordance with the approved guidelines of the IZW.

### Blood sampling and storage

Blood was taken with serum and heparin Vacutainer tubes (Becton Dickinson, Franklin Lakes, USA), transported to the field laboratory in a cool box and centrifuged within 12 hours, very rarely within 24 hours after sampling. Results of immunological tests conducted with serum and plasma from whole blood centrifuged after 2 hours, 4 hours, 6 hours and 12 hours from blood collection and after 2 hours and 4 hours from blood collection, respectively, do not differ^100^. There was no systematic bias for samples centrifuged later than 12 h after sampling, thus all samples were included for immunological tests. Serum and plasma were aliquoted and stored in liquid nitrogen. Samples were transported to Germany in full compliance with the Convention on International Trade in Endangered Species (CITES) and stored at −80 °C until laboratory analysis.

### ELISA for Immunoglobulin G

Immunoglobulin G (IgG) concentration were measured in plasma samples with a protein A enzyme-linked immunosorbent assay (ELISA)^101^. Plasma samples were diluted 1:20.000 with 50 mM NaHCO_3_. As a standard, we diluted purified cat IgG with a starting concentration of 1 mg/ml (Bethyl Laboratories, Montgomery, USA, Catalog N° P20-105) with 50 mM NaHCO_3_ and created standard concentrations of 4.0 μg/ml, 2.0 μg/ml, 1.0 μg/ml, 0.5 μg/ml, 0.25 μg/ml, 0.125 μg/ml and 0.0625 μg/ml.

We pipetted 100 μl of diluted samples or standards in duplicates into each well of 96-well ELISA plates. Plates were incubated for 1 hour at 37 °C and washed twice with Tris-Buffered-Saline-Tween-20 (TSB-T20). Gelatine was added to TSB-T20 solution and 200 μl of 1% of this mixture was pipetted to each well to block non-specific reaction bindings. Plates were incubated for 30 min at 37 °C and washed twice with TSB-T20. Then, 100 μl of 1:12.000 solution of protein-A-horseradish-peroxidase (Protein A- HRP; Invitrogen; Catalog N° 10-1023) in TSB-T20 was added to each well and plates were incubated for 30 min at room temperature. Plates were washed with TSB-T20 and 100 μl of phosphate-citrate-buffer containing a 1% dilution of 3,3′,5,5′-Tetramethylbenzidin (TMB; TMB One Component Microwell Substrate; SouthernBiotech; Catalog N° 0411-01), dimethylsulfoxide (DMSO) and H_2_O_2_ was added to each well. The reaction was stopped after 5 min with 100 μl of 1% H_2_SO_4_. We measured the absorbance of the wells in the plates at 450 nm in a photometric microplate reader (Biotek; μQuant Microplate Spectrophotometer). A linear standard curve was calculated for each plate using the standard concentrations. IgG concentrations were then calculated based on the standard curve.

### ELISA for serum amyloid A (SAA)

We measured and calculated SAA concentrations with a commercial solid phase sandwich SAA Multispecies ELISA kit (Tridelta, Phase Range; Multispecies SAA ELISA kit; Catalog N° TP-802) following the instructions of the manufacturer. Cheetah and leopard plasma samples were diluted 1:1,500 and 1:2,000, respectively, with sample diluent (provided with the ELISA kit) prior to the assay. A linear standard curve was calculated using the calibrator standard for cats (100.0 ng/ml, 50.0 ng/ml, 25.0 ng/ml, 12.5 ng/ml, 6.25 ng/ml and 0.0 ng/ml). SAA concentrations were calculated according to the standard curve on each plate. Sixty-two samples had a higher absorbance than measurable with our spectrophotometer (Biotek; μQuant Microplate Spectrophotometer). These samples were either rerun (*N* = 42) with a higher dilution of 1:5,000 for cheetahs or 1:8,000 for leopards (samples were thawed again) or the entire plate was re-measured directly after discarding half of the volume in each well (*N* = 20).

### Bacterial killing assay (BKA)

We measured the *in vitro* bacterial killing ability of serum against *Escherichia coli*. The method was previously described in detail in ref. 102. Briefly, serum samples were serially diluted with phosphate-buffered-saline (PBS), resulting in eight dilutions from 1:2 to 1:265. Each well of a 96-well-plate was filled with 44 μl and mixed with 10 μl of a bacterial working solution of ~1.5 × 10^5^ colony-forming units (CFU)/ml. After incubation for 30 min at 37 °C, tryptic soy broth was added to each well. Absorbance was measured with a spectrophotometer (Biotek; μQuant Microplate Spectrophotometer) to determine background absorbance and again after the plates had been incubated for 12 hours at 37 °C. Bacterial killing capacity was calculated for each dilution of serum against a positive control (wells that contain only bacteria without serum). Ranks were assigned to each dilution before killing capacity dropped from 100% to 0%, such that dilution 1:2 corresponded to rank 1, the usually lowest rank, dilution 1:4 to rank 2, etc. If bacterial killing did not reach 100% even at dilution 1:2, rank 0 was assigned.

### Lysoplate assay

To measure the concentration of lysozyme we used the lysoplate assay method^103^. We prepared 1% noble agar (Sigma Aldrich; St. Louis, USA, Catalog N° A5431-250G) with PBS at pH = 6.3 and added the required amount of lysozyme-sensitive bacteria *Micrococcus lysodeikticus* (Sigma Aldrich; M3770) to reach a bacterial concentration of 25 mg/100 ml in the agar for cheetahs and 12.5 mg/100 ml in the agar for leopards. Plates had a diameter of 14.2 cm and were put on a pre-heated surface (50 °C), horizontally leveled with a water spirit to avoid quick and uneven cooling of the 30 ml noble agar on the plates. After cooling, 25 holes with diameters of 4.5 mm were punched into the agar and filled with 25 μl of serum samples (18 holes) or standards (7 holes). Standards with concentrations of 10.0 μg/ml, 7.5 μg/ml, 5.0 μg/ml, 2.5 μg/ml, 2.0 μg/ml, 1.25 μg/ml and 1.0 μg/ml were prepared using lysozyme form chicken egg white (Sigma Aldrich; St. Louis, USA, Catalog N° L6876). Plates were incubated at room temperature for 18 hours.

*Micrococcus lysodeikticus* is particularly sensitive to lysozyme, thus the bacterial lysis of the samples and standards creates a clear zone around the inoculated wells. The diameter of this clear zone is proportional to the logarithmic (basis of 10) lysozyme concentration in the samples and standards^103^. We photographed each plate in a photobox (Imaging system; peqlab) with a ruler next to it as a reference scale. The diameter of the lytic areas was measured digitally using the software ImageJ (version 1.48, http://imagej.nih.gov/ij/). Each lytic area was measured three times and the mean was used for calculations. The measurements of the lysis standards were plotted as a linear function of the log lysozyme concentration. This regression line was then used to infer the lysozyme concentrations of the cheetah and leopard samples.

### Haemagglutination/haemolysis assay

The haemagglutination/haemolysis titers represent the levels of natural antibodies and complement^61^. Although the method was originally developed for avian species, it has recently been modified for mammals by using chicken erythrocytes as target cells^62^. After pipetting 25 μl of plasma in the wells of the first two columns of a U-shaped 96-well microtitre plate, 25 μl sterile PBS was added to the 2^nd^–12^th^ columns. Using a multi-channel pipette, the content of the second column wells was serially diluted until the 11^th^ column, resulting in a dilution series for each sample from 1:2 to 1:1024. We used the last column of the plate as negative controls containing only PBS. We then added 25 μl of 1% chicken red blood cells suspension to all wells, covered them with Parafilm M (Pechiney Plastic Packaging, Chicago, USA), vortexed gently and incubated at 37 °C for 90 min. After incubation the plates were tilted at a 45° angle to increase the visualization of agglutination and kept at room temperature until analyses.

Agglutination and lysis, which reflect the activity of natural antibodies and the interaction between natural antibodies and complement^61^,^104^, were recorded after 20 min (haemagglutination titre) and 90 min (haemolysis titre), respectively. Haemagglutination is characterized by the appearance of clumped red blood cells as a result of antibodies binding multiple antigens, whereas during haemolysis red blood cells are destroyed by complement. Haemagglutination/haemolysis titers were given as the log_2_ of the reciprocal of the highest dilution (i.e. lowest concentration) of plasma showing positive haemagglutination or haemolysis, respectively^62^,^104^.

### Measurement of cortisol concentration

Although cheetahs and leopards were captured in the same type of traps and therefore exposed to the same capture conditions, the two species might respond differently to these short-term challenges. Such challenges increase the allostastic load (‘stress’) and thereby may influence various immune parameters, as has been shown for SAA concentrations in rats^53^. To rule out the possibility that differences in immune parameters between the two species were caused by differences in allostatic load induced by different responses to the capture procedure, we measured the concentration of native cortisol, an indicator of allostatic load which rapidly increases after a stressful stimulus^105^, in blood samples of cheetahs and leopards. Cortisol (hydrocortisone) was quantified as described earlier^106^ by an enzyme immunoassay (EIA) using a polyclonal antibody (rabbit) against hydrocortisone-21-hemisuccinate-BSA and hydrocortisone-21-hemisuccinate-peroxidase as label. The inter-assay coefficient of variation of two biological samples was 7.3 and 8.1% (n = 14), respectively.

### Statistical analyses

The dataset consisted of 251 captures and sampling events for 197 cheetahs and 36 captures and sampling events for 36 leopards. Sample sizes varied slightly for different immunological measurements because the bacterial killing assay and the hemagglutination/hemolysis assay should be limited to samples which are thawed for the first time only, otherwise they become unreliable. Some samples had been previously thawed for other studies and been frozen again and therefore could not be used. Other samples were too small to provide material for all analyses. We therefore expect that there was no systematic bias in the sets of available samples for the analysis of various immune parameters of cheetahs and leopards. The highest number of missing data was for SAA concentration with 68 missing measurements (26.8%). For all other immunological parameters the percentage of missing measurements was below 20%. We used principal component analysis (PCA), a statistical procedure that uses an orthogonal transformation to convert a set of possibly correlated variables into a set of linearly uncorrelated variables. PCA reduces the dimensionality of data and through the loadings of the original variables on the principal components helps to identify the true sources of variation in the data. Because PCA requires the removal of all samples with missing data, we used the R package Amelia II version 1.7.3, which allows for the imputation of missing values in the dataset^107^. Amelia II uses the expectation-maximization algorithm to perform multiple imputations for incomplete data. An examination of the quality of imputation is presented in the supplementary information (Figure S1, Table S1 and Supplementary Results).

To test whether both species differ in their immune characteristics, we used the dataset complemented by the imputed values and reduced the dimensionality of immune parameters by performing a mean-centered, scaled PCA with the R package ade4 version 1.7-2^108^. We then fitted a logistic regression model predicting species identity (a binary variable) as a function of the first two principal components. We tested the effect of each covariate by performing a likelihood ratio test between this model and a model that only contained the intercept and the other principal component. We ruled out the possibility that differences between species were a consequence of possible differences in the proportion of males and females sampled in the two species. To do so, we fitted one logistic regression per species predicting the sex (binary variable) as a function of the two principal components of the PCA, and compared the likelihood of the fit to a model with an intercept only. A non-significant result of this analysis would suggest that sex does not influence the outcome of the PCA analysis which uses the immune components to distinguish species. To control for the possible effects of allostatic load we fitted linear models predicting each immune variable by cortisol concentrations to extract the residuals of these models to be used in another PCA. We again predicted the species identity (binary variable) as a function of the two principal components from this new PCA with a logistic regression model and compared the results obtained with those from the first PCA. All statistical analyses were performed using R version 3.0.3^109^.

Following the PCA analyses, we compared the median of each immune parameter separately with non-parametric Mann-Whitney-U-tests for not normally distributed data. This allowed us to use the original non-imputed dataset. Levels of injuries caused by the behavior of the animals in the box traps were analyzed with a chi-square-test for independence.

## Additional Information

**How to cite this article:** Heinrich, S. K. *et al*. Cheetahs have a stronger constitutive innate immunity than leopards. *Sci. Rep.* **7**, 44837; doi: 10.1038/srep44837 (2017).

**Publisher's note:** Springer Nature remains neutral with regard to jurisdictional claims in published maps and institutional affiliations.

## Supplementary Material

Supplementary Figure and Tables
