# Supplementary material for: Cheetahs have a stronger constitutive innate immunity than leopards
Source: Sci Rep. 2017 Mar 23;7:44837. doi: 10.1038/srep44837 (PMC5363065; doi:10.1038/srep44837)
Supplement: Supplementary Figure and Tables [file srep44837-s1.doc]

# Cheetahs compensate low major histocompatibility complex variability with a strong innate immune system

# Electronic supplementary material

Sonja K. Heinrich, Heribert Hofer, Alexandre Courtiol, Jörg Melzheimer, Martin Dehnhard, Gábor Á. Czirják*, Bettina Wachter*

**1. Supplementary methods: Statistical analyses p2**

Figure S1 p3

Table S1 p4

**2. Supplementary results: distinguishing species p5**

Table S2 p5

**3. Supplementary results: the effect of imputation p6**

**Figure S2 p7**

4. Supplementary results: the effect of allostatic load………………………………………...p7

**Table S3 p8**

**5. Supplementary results: the combined effect of imputation and allostatic load p8**

**Table S4 p9**

# 1. Supplementary methods: Statistical analyses

Missing data in our dataset were imputed with the R package Amelia II version 1.7.3 [^1^](#_ENREF_1). Amelia II uses the expectation-maximization algorithm to perform imputation for incomplete data. Because our dataset had missing values amongst all immune variables, we used the method of multiple imputation to replace missing values and thereby increase the power of the statistical models.

Figure S1 presents a comparison of the observed, original dataset with the dataset which included imputed values. During imputation, BKA, haemagglutination and haemolysis were considered as continuous variables in order to improve imputation performance, which explains some differences in the distribution between original and imputed data (Fig. S1). Table S1 presents the results of the statistical comparison of imputed and original data, using Mann-Whitney U-tests for differences in the median of both data sets and Levene’s test for differences in the variance.

To correct results for differences in allostatic load, we performed a linear model predicting each immune variable from glucocorticoid concentrations. We then extracted the residuals of these linear models to obtain the variation in allostatic load that is unrelated to variation in glucocorticoid concentrations. The residual variation associated with each immune variable constituted a set of new variables, which were used for another principal component analysis (PCA). The new principal component 1 (PC1) and principal component 2 (PC2) derived from this PCA were then used as input parameters for alternative models.

**Figure S1 Comparison of distributions of observed and imputed values for all immune variables. The observed values are drawn in black, imputed* values in red.**

*****Imputation is the process of replacing missing data with substituted values. The R package “Amelia II”^1^ implements a bootstrapping-based algorithm for multiple imputation of missing values.


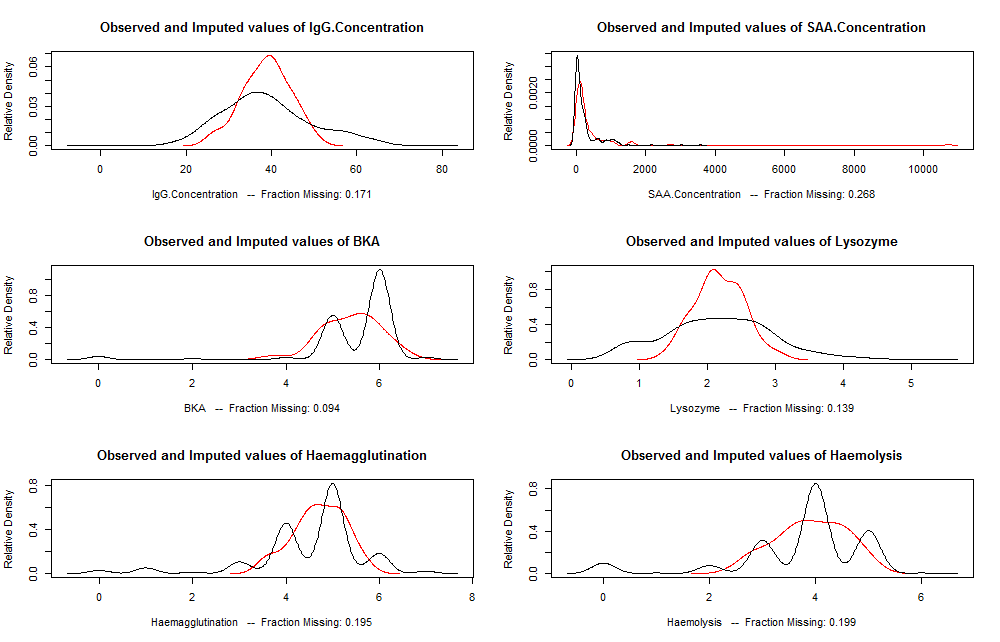


| **Table S1 Comparison of original data and imputed data.** | | | | | | | |
| --- | --- | --- | --- | --- | --- | --- | --- |
| **Variable** | **Mann-Whitney U-test** | | **Median original data** | **Median imputed data** | **Levene’s test** | **variance original data** | **variance imputed data** |
| IgG concentration^a^ | | W=5433; p=0.452 | 37.45 | 37.29 | F=19.35; p<0.001 | 118.70 | 26.75 |
| SAA concentration^b^ | | W=9779, p=0.007 | 86.77 | 130.49 | F=1.53; p=0.21 | 2026631 | 210465.2 |
| BKA rank^c^ | | W=3596; p=0.816 | 6.00 | 5.33 | F=0.10; p=0.75 | 1.20 | 0.35 |
| Lysozyme concentration | | W=4698; p=0.620 | 2.16 | 2.16 | F=16.09; p<0.001 | 0.63 | 0.15 |
| Haemagglutination titer | | W=6874; p=0.453 | 5.00 | 4.76 | F=8.10; p=0.005 | 1.44 | 0.22 |
| Haemolysis titer | | W=7187; p=0.244 | 4.00 | 4.10 | F=4.88; p=0.028 | 1.48 | 0.33 |

^a^IgG: Immunoglobulin G

^b^SAA: Serum Amyloid A

^c^BKA: Bacterial killing assay

# The PCAs using the imputed dataset (1) without correction for allostatic load provided the basis for Model 1, described in the main text, with detailed factor loadings specified in Table S3, and (2) with correction for allostatic load provided the basis for Model 2, described below, with detailed factor loadings specified in Table S3. The PCAs using the smaller datset of observed, original data without imputation (1) without correction for allostatic load provided the basis for Model 3, described below, with detailed factor loadings specified in Table S4, and (2) with correction for allostatic load provided the basis for Model 4, described below, with detailed factor loadings specified in Table S4.

# 2. Supplementary results: distinguishing species

Table S2 presents the results of a logistic regression to check whether the two species can be reliably distinguished by the results of the PCA performed on imputed data (Model 1).

| **Table S2 │** **Summary table of the logistic regression model (Model 1) predicting whether the species is a cheetah (value = 1) or a leopard (value = 0). Estimates and associated standard error are expressed on the logit scale. P-values are here computed using a simple z-test on the estimates. See main text for likelihood-ratio tests.** | | | | |
| --- | --- | --- | --- | --- |
|  | **Estimate** | **Standard error** | **z-value** | **p-value** |
| Intercept | 2.811 | 0.364 | 7.715 | <0.001 |
| PC1^a^ | 0.389 | 0.194 | 2.007 | 0.045 |
| PC2 | -2.273 | 0.368 | -6.170 | <0.001 |

^a^PC: Principal component

# 3. Supplementary results: the effect of imputation

Imputation significantly shifted (increased) the median only in the case of SAA concentrations but not for the other immunology variables (Table S1). Imputation also reduced the variance from the original data for all immunology variables except SAA concentration, and reduced it significantly for IgG concentration, haemagglutination titer, haemolysis titer and lysozyme concentration (Table S1). A lower variance reduces the sensitivity of our analysis to imputed values, which makes the analysis therefore conservative.

Figure S2 presents the results of a PCA on the raw dataset, i.e. without imputing missing values (*N_cheetahs_* = 80, *N_leopards_* = 29). The results were qualitatively similar. Here PC1 captured 43.4% of the total variance in immune measurements (as compared to 40.1% with imputed data), PC2 captured 22.6% of the total variance (as compared to 23.8% with imputed data). The detailed factor loadings are listed in Table S4. In the logistic regression model for this non-imputed dataset (Model 3), cheetahs presented higher, but not significantly higher, scores on PC1 (mean PC1 value = 0.046) than leopards (mean PC1 value = -0.127, logistic regression, likelihood ratio test (LRT) = 0.42, *df* =1, *P* =0.51) and significantly lower scores on PC2 (mean PC2 value = -0.375) than leopards (mean PC2 value = 1.035, logistic regression, LRT = 55.65, *df* = 1, *P* < 0.001). In comparison, with imputed data, cheetahs presented significantly higher scores on PC1 and significantly lower scores on PC2 than leopards (see main text).

**Figure S2 Immune differences between cheetahs and leopards based on original (non-imputed) data (Model 3).** Scores of all cheetah (small circles) and leopard individuals (plus signs) on the first two principal components (PC1 on x-axis and PC2 on y-axis) of a principal component analysis performed on all six immune parameters. Arrows represent the contribution of each immune parameter to PC1 and PC2. For each species, 1.5 inertia ellipses are depicted.


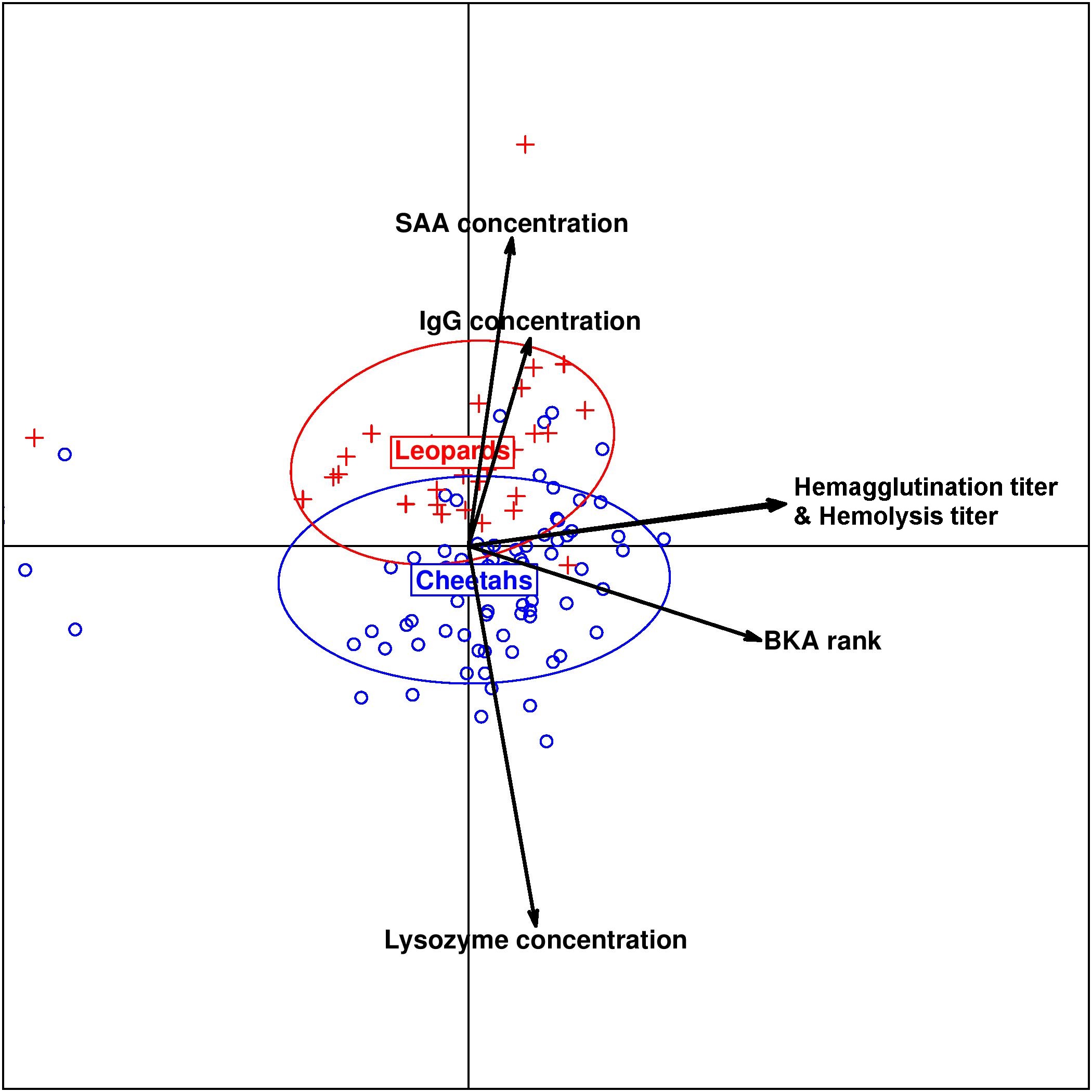


# 4 Supplementary results: the importance of allostatic load

Table S3 specifies the detailed factor loadings for each original immune variable on the PCs for both the original model (Model 1) and the alternative model (Model 2) where we accounted for the variation in cortisol concentration between species. Note that both models used imputed data. The strongest shift in loadings affected the SAA concentration (halving the loading on PC1 and thereby reducing its impact on PC1), followed by an increase in the loading of IgG concentration on PC2 and a decrease of the loading of BKA rank on PC2, each by approximately a third of the original value.

| **Table S3 PCA loadings for Principal Component 1 and 2 using imputed data** | | | | |
| --- | --- | --- | --- | --- |
| **Immune variable** | **Principal component 1** | | **Principal component 2** | |
|  | **Model 1** | **Model 2** | **Model 1** | **Model 2** |
| Correcting for  allostatic load? | No | Yes | No | Yes |
| IgG concentration^a^ | 0.2318 | 0.222 | 0.2935 | 0.3824 |
| SAA concentration^b^ | 0.2991 | 0.1571 | 0.7578 | 0.7450 |
| BKA rank^c^ | 0.7652 | 0.7693 | -0.0799 | 0.0016 |
| Lysozyme concentration | 0.3005 | 0.3744 | -0.7946 | -0.7174 |
| Haemagglutination titer | 0.9170 | 0.9208 | 0.0243 | 0.0093 |
| Haemolysis titer | 0.9273 | 0.9227 | 0.0296 | 0.0615 |

^a^IgG: Immunoglobulin G

^b^SAA: Serum Amyloid A

^c^BKA: Bacterial killing assay

# 5 Supplementary results: the combined effect of imputation and of allostatic load

Table S4 specifies the results from two PCAs of the observed, original dataset without or with correction for allostatic load, by listing detailed factor loadings for each original immune variable. The main difference between the two PCAs is the increase in the loading of SAA concentrations on PC1, otherwise the loadings are very similar between both PCAs. The results from these PCAs are qualitatively similar to the respective PCAs run on the imputed data set (Table S3), in that the relative contribution of the original immune variables to PC1 and PC2 were preserved, even if in the imputed data set some of the loadings were stronger.

In a logistic regression model of the PCA using the non-imputed dataset (*N_cheetahs_* = 80, *N_leopards_* = 29) corrected for allostatic load (Model 4, depicted in Table S4), PC1 was not significantly different between cheetahs (mean PC1 value = 0.039) and leopards (mean PC1 value = -0.108) (logistic regression predicting the species from PC values; LRT = 0.41, *df* = 1, *P* = 0.522). There was a significant difference between cheetahs (mean PC2 value = -0.168) and leopards (mean PC2 value = 0.463) for PC2 (logistic regression predicting the species from PC values; LRT = 9.38, *df* = 1, *P* = 0.002). For this PCA (Model 4), PC1 captured 44.1% of the total variance in immune measurements and PC2 captured 23.1%.

| **Table S4 PCA loadings for Principal Component 1 and 2 using non- imputed data** | | | | |
| --- | --- | --- | --- | --- |
| **Immune variable** | **Principal component 1** | | **Principal component 2** | |
|  | **Model 3** | **Model 4** | **Model 3** | **Model 4** |
| Correcting for  allostatic load? | No | Yes | No | Yes |
| IgG concentration^a^ | 0.1836 | 0.1751 | 0.4446 | 0.4581 |
| SAA concentration^b^ | 0.1294 | 0.1899 | 0.6602 | 0.6817 |
| BKA rank^c^ | 0.8690 | 0.8719 | -0.2021 | -0.1605 |
| Lysozyme concentration | 0.2005 | 0.2359 | -0.8151 | -0.8235 |
| Haemagglutination titer | 0.9407 | 0.9395 | 0.0890 | 0.0618 |
| Haemolysis titer | 0.9354 | 0.9370 | 0.0944 | 0.0710 |

^a^IgG: Immunoglobulin G

^b^SAA: Serum Amyloid A

^c^BKA: Bacterial killing assay
